# SARS-CoV-2 infection in children: A 24 months experience with focus on risk factors in a pediatric tertiary care hospital in Milan, Italy

**DOI:** 10.3389/fped.2023.1082083

**Published:** 2023-02-17

**Authors:** Giada Maria Di Pietro, Luisa Ronzoni, Lorenzo Maria Meschia, Claudia Tagliabue, Angela Lombardi, Raffaella Pinzani, Samantha Bosis, Paola Giovanna Marchisio, Luca Valenti

**Affiliations:** ^1^ S.C. Pediatria—Pneumoinfettivologia, Fondazione IRCCS Ca’ Granda Ospedale Maggiore Policlinico, Milan, Italy; ^2^ Precision Medicine Lab, Transfusion Medicine and Hematology, Biological Resource Centre, Fondazione IRCCS Ca’ Granda Ospedale Maggiore Policlinico, Milan, Italy; ^3^ Department of Pathophysiology and Transplantation, Università Degli Studi di Milano, Milan, Italy

**Keywords:** SARS-CoV-2 infection, children, COVID-19, risk factors, complications, clinical characteristics, genetic predisposition, OAS3 2

## Abstract

**Background:**

Severe acute respiratory syndrome coronavirus 2 (SARS-CoV-2) infection in children is characterized by a wide variety of expressions ranging from asymptomatic to, rarely, critical illness. The basis of this variability is not yet fully understood. The aim of this study was to identify clinical and genetic risk factors predisposing to disease susceptibility and progression in children.

**Methods:**

We enrolled 181 consecutive children aged less than 18 years hospitalized with or for SARS-CoV-2 infection during a period of 24 months. Demographic, clinical, laboratory, and microbiological data were collected. The development of coronavirus disease 2019 (COVID-19)-related complications and their specific therapies were assessed. In a subset of 79 children, a genetic analysis was carried out to evaluate the role of common COVID-19 genetic risk factors (chromosome 3 cluster; *ABO*-blood group system; *FUT2*, *IFNAR2*, *OAS1/2/3*, and *DPP9* loci).

**Results:**

The mean age of hospitalized children was 5.7 years, 30.9% of them being under 1 year of age. The majority of children (63%) were hospitalized for reasons different than COVID-19 and incidentally tested positive for SARS-CoV-2, while 37% were admitted for SARS-CoV-2 infection. Chronic underlying diseases were reported in 29.8% of children. The majority of children were asymptomatic or mildly symptomatic; only 12.7% developed a moderate to critical disease. A concomitant pathogen, mainly respiratory viruses, was isolated in 53.3%. Complications were reported in 7% of children admitted for other reasons and in 28.3% of those hospitalized for COVID-19. The respiratory system was most frequently involved, and the C-reactive protein was the laboratory test most related to the development of critical clinical complications. The main risk factors for complication development were prematurity [relative risk (RR) 3.8, 95% confidence interval (CI) 2.4–6.1], comorbidities (RR 4.5, 95% CI 3.3–5.6), and the presence of coinfections (RR 2.5, 95% CI 1.1–5.75). The *OAS1/2/3* risk variant was the main genetic risk factor for pneumonia development [Odds ratio (OR) 3.28, 95% CI 1-10.7; *p* value 0.049].

**Conclusion:**

Our study confirmed that COVID-19 is generally less severe in children, although complications can develop, especially in those with comorbidities (chronic diseases or prematurity) and coinfections. Variation at the *OAS1/2/3* genes cluster is the main genetic risk factor predisposing to COVID-19 pneumonia in children.

## Introduction

At the end of 2019, a novel Coronavirus was identified as the cause of a cluster of pneumonia cases in Wuhan, a city in the Hubei province of China. The virus responsible for the coronavirus disease, known as coronavirus disease 2019 (COVID-19), was designated as severe acute respiratory syndrome coronavirus 2 (SARS-CoV-2) (1). It quickly spread around the world and the World Health Organization (WHO) declared in March 2020 that the COVID-19 outbreak was a global pandemic (2). SARS-CoV-2 infection is characterized by a wide variety of clinical expressions ranging from no symptoms to severe and potentially lethal acute respiratory distress syndrome (ARDS), mainly in adults. The natural history of the SARS-COV-2 infection, especially in those who developed complications, is characterized by a first viral replication phase with mild to moderate symptoms, followed by a second pulmonary stage with dyspnea and consequently hypoxia. If antiviral treatment is promptly set up, the patient can improve, but sometimes there is a progression to a third stage, with an hyperinflammatory status due to a cytokine storm that rapidly evolved in worsening of general conditions with the necessity of mechanical ventilation resulting in a very high mortality. Although COVID-19 illness is generally milder in children when compared with adults, with low risks of hospitalization and death, a small minority of children develop complications or present with a multisystem inflammatory state with an associated mortality rate of 1%–2% (3–10). The likelihood of evolving to a severe COVID-19 is higher in those with comorbidities or with a concomitant infection. Children affected by chronic diseases such as obesity, diabetes, heart or lung diseases, neurologic disorders, or with underlying clinical conditions such as congenital malformations, complex syndromes, or with an immunocompromised status are at higher risk of severe COVID-19. Furthermore, concomitant infecting pathogens are another factor contributing to the severity of COVID-19: microbial coinfections have been associated with a major probability of intensive care and ventilator support; an early diagnosis is crucial for prompt intervention and better management and prognosis of COVID infection (11–13). Host genetic factors contributing to infection susceptibility and disease severity have been largely explored in adults by genome-wide association studies (GWAS). The strongest and most consistent signals were identified at chromosome 3p21.31 spanning numerous genes including *LZTFL1*; variants at these loci are associated with increased risks of morbidity and mortality, which are more pronounced among individuals aged 60 years or younger (14–16). Furthermore, the *ABO*-blood group system has been associated with susceptibility to SARS-CoV-2 infection with the non-O blood group predisposing to COVID-19 infection and the *ABO* non-secretor phenotype (encoded by *FUT2* gene) with protection against severe disease (15, 17, 18). Several other genome-wide associations have been identified, including genes encoding the antiviral restriction enzyme activators (at the *OAS1–OAS2–OAS3* gene cluster) whose mutations correlate with lower innate antiviral defenses, the gene encoding dipeptidyl peptidase 9 (*DPP9*) whose mutation predisposes to a higher inflammatory lung injury and the interferon receptor gene (*IFNAR2*) whose low expression is associated with life-threatening disease (19–21). The pathogenesis of COVID-19 in children and the basis of its clinical variability are not yet fully understood, and most of the available information is currently extrapolated from genetic findings in adults. The purpose of our study was, therefore, to evaluate the risk of progression to severe disease in a cohort of consecutive children hospitalized for COVID-19 at a referral institution, to examine the risk factors predisposing this evolution, and to evaluate, among children hospitalized for reasons other than COVID-19 but with an incidental positivity to SARS-CoV-2, whether the presence of the infection would have increased the risk of developing complications. Moreover, in a subset of infected hospitalized children, we evaluated the predisposing genetic loci identified in adults to assess the impact of these factors on disease susceptibility and progression in children.

## Materials and methods

### Patient cohort

The study was conducted in a tertiary care Hospital (Fondazione IRCCS Cà Granda Ospedale Maggiore Policlinico) in Milan, Northern Italy. All subjects younger than 18 years admitted to the Pediatrics Clinic with documented SARS-CoV-2 infection were enrolled. The recruitment took place from March 1, 2020, to March 31, 2022. Diagnosis of SARS-CoV-2 infection was established in presence of at least one nasopharyngeal aspirate or swab positive for SARS-CoV-2 RNA. Specimens were collected through nasopharyngeal aspirate for children ≤6 years of age, while a nasopharyngeal swab was used for those older than 6 years with. SARS-CoV-2 nucleic acid testing was performed by real-time reverse-transcriptase polymerase-chain-reaction (RT-PCR). We considered positive all samples in which the following genes were detected: RNA-polymerase-RNA-dependent, nucleocapsid gene (N) and gene envelope (E) and with a cycle threshold (CT) value <40 (22). Exclusion criteria were being older than 18 years at the time of admission and to have a negative SARS-CoV-2 nasopharyngeal swab. Children with a diagnosis of “multisystem inflammatory syndrome” were excluded because this condition usually occurs in those who have had a history of COVID-19 while our study focused on acute SARS-CoV-2 positivity. The population was divided into two groups: those admitted for other reasons with an incidental diagnosis of SARS-CoV-2 infection and those hospitalized for COVID-19. The first group included all children admitted continuing chronic therapies or to treat relapses of chronic diseases, to perform planned diagnostic tests, for social problems, psychiatric diseases, trauma or fractures, and for diseases not related to SARS-CoV-2 infection (urinary tract infections or pyelonephritis, arthritis, osteomyelitis). All patients with symptoms due to SARS-CoV-2 infection belonged to the second group.

The demographic, clinical, and laboratory data of each patient were reported in an electronic database. Data collection was allowed by the written consent of at least one parent. For each patient, we collected data regarding the gender, date of birth, age at admission, ethnicity, preexisting chronic diseases and chronic therapies, and COVID-19 vaccination status. Whenever possible, the source of infection (family, community) was investigated. The symptoms’ onset, the reason for the admission to the hospital, and the development of COVID-19-related complications were recorded. COVID-19-related complications were defined as follows: clinical and/or radiological diagnosis of pneumonia; severe acute respiratory illness or SARI (at least one respiratory sign/symptom on admission and at least one systemic sign/symptom in the previous 7 days or worsening of the clinical conditions during hospitalization); acute respiratory distress syndrome or ARDS (characterized by hypoxemia with bilateral pulmonary infiltrates compatible with non-cardiogenic edema). Those children affected by pneumonia or those who evolved to SARI belonged to moderate/severe forms, while ARDS was defined as critical diseases. We also investigated the need for care provided in the pediatric intensive care unit (PICU), the requirement for noninvasive ventilation or mechanical ventilation, and extracorporeal membrane oxygenation (ECMO). We also collected results of blood tests [blood count, C-reactive protein (CRP), coagulation, liver and kidney function, ferritin values], radiological examinations (chest x-ray, chest CT, and ultrasound examinations when performed), and the presence of coinfections. We recorded the results of tests performed to identify viruses and bacteria on blood (by culture, specific antibody, or PCR amplification), on feces and urine, and on nasopharyngeal aspirates (obtained in children with and without respiratory symptoms). Finally, the length of stay, therapies administered for COVID-19 infection (antiviral and monoclonal antibodies), and the persistence of long-term sequelae were assessed.

### Genetic analysis

After obtaining written informed consent from at least one parent, a blood sample was collected from 79 hospitalized infected children, who consented also to genetic analysis, in order to evaluate the contribution of genetic factors to the clinical variability of COVID-19. DNA was extracted from peripheral blood by the QIASymphony (Qiagen, Milan, Italy). Genotyping for common genetic risk factors for COVID-19 susceptibility or severity in adult patients (*LZTFL1* rs11385942 tagging chromosome 3 cluster, *OAS3* rs10735079 tagging the *OAS1/2/3* locus variability, *FUT2* rs601338, *IFNAR2* rs2229207, and *DPP9* rs2109069) was carried out using the TaqMan SNP genotyping allelic discrimination method (Applied Biosystems). Commercial genotyping assays were available. Genotypes were analyzed by the SDS software v.2.3 (StepOne Plus; Applied Biosystems). *ABO* genetic locus variation was determined using the rs657152, as previously described (14–20). The frequencies of these variants observed in children were compared to that of a control cohort (2,848 healthy subjects) and an adult cohort with severe COVID-19 (995 patients) (23). The genetic study was approved by the Ethics Committee of the Fondazione IRCCS Ca’ Granda (FoGS, approval number 342_2020).

### Statistical analysis

Categorical variables were expressed as frequencies and percentages; continuous variables were presented as mean and standard deviation (SD) or median and interquartile range (IQR), as appropriate. Continuous variables were compared between groups using Wilcoxon Rank-Sum nonparametric test or Student's-*t*-test. Categorical variables were analyzed using Chi-square test. The children admitted for COVID-19 was divided into two categories, those with complicated COVID-19 (CC group) and those with noncomplicated COVID-19 (NC group). CRP values expressed in mg/dL of the two groups were compared using Wilcoxon Rank-Sum nonparametric test. Possible risk factors for progression to complicated forms were analyzed, estimating their impact by relative risk (RR) and 95% confidence intervals (CIs), by comparison between complicated patients and noncomplicated patients.

Univariate logistic regression models were used to compare genetic risk variants frequency between children cohort, COVID-19 adult cohort, and control cohort, under an additive genetic model. Univariate and multiple logistic regression models were used to assess clinical and genetic factors associated with the development of SARS-CoV-2 complications. Statistical analysis was carried out using the JMP Pro 16.0 Statistical Analysis Software (SAS Institute, Cary, NC, United States). *p* values <0.05 (two tailed) were considered significant.

## Results

### Study population

A total of 181 children and adolescents <18 years of age (mean age 5.7 years, 95% CI 4.83–6.57) were enrolled: 26 were hospitalized between March 1, 2020, and July 31, 2020; 34 between September 1, 2020, and December 31, 2020; 42 between January 1 2021, and July 31, 2021; and 79 between September 1, 2021, and March 31, 2022. The majority of children (114/181; 63%) were hospitalized for reasons different from COVID-19 (Supplementary Figure S1), while 67/181 (37%) were admitted due to SARS-CoV-2 infection. Demographic characteristics are shown in Table 1. About 30% (54/181) of the patients suffered from chronic diseases, 77% of whom (42/54) required chronic therapies. The main comorbidities were the presence of kidney diseases or congenital anomalies. Seventy-four/181 (40.8%) children have had contact with a family member positive for SARS-CoV-2; 58/74 had at least one infected parent; among the latter both parents infected were reported in the 36.2% (21/58). Of children without a history of close contact with an infected relative, 34/107 had however a parent who tested positive at the time of admission.

**Table 1:** Demographic characteristics of study population.

	Total (*N* = 181)	Children hospitalized for COVID-19 (*N* = 67)	Children admitted for other reasons with incidental diagnosis of SARS-CoV-2 infection (*N* = 114)
Children age (years), median (IQR)	3 (0–11)	3 (0–10)	3 (0–11)
Age category (years), *n* (%)			
<1	56 (30.9)	31 (46.3)	25 (21.9)
<1 and ≤6	55 (30.3)	23 (34.3)	32 (28.1)
<7 and ≤10	23 (12.7)	3 (4.5)	20 (17.5)
>11	47 (25.9)	10 (14.9)	37 (32.5)
Gender, *n* (%)			
Males	101 (55.8)	38 (56.7)	63 (55.3)
Females	80 (44.2)	29 (43.3)	51 (44.7)
Ethnicity, *n* (%)			
Caucasian	129 (71.2)	50 (74.6)	79 (69.3)
Not Caucasian	52 (28.8)	17 (25.4)	35 (30.7)
Prematurity, yes, *n* (%)	13 (7.1)	4 (6.0)	9 (7.9)
Underlying chronic diseases, yes, *n* (%)	54 (29.8)	19 (28.4)	35 (30.7)
Kidney diseases	13 (24.1)	5 (26.3)	8 (22.9)
Comorbidities related to prematurity	4 (7.4)	3 (15.8)	1 (2.9)
Rheumatologic diseases	4 (7.4)	0	4 (11.4)
Congenital malformations/syndromes	16 (29.6)	4 (21.1)	12 (34.3)
Cystic fibrosis	2 (3.7)	1 (5.3)	1 (2.9)
Asthma	2 (3.7)	0	2 (5.7)
Psychiatric disorders	3 (5.6)	0	3 (8.6)
Miscellaneous	10 (18.5)	6 (31.6)	4 (11.4)

SARS-CoV-2, severe acute respiratory syndrome coronavirus 2; COVID-19, coronavirus disease 2019.

The first number is the absolute value, while the second, in brackets, is the associated percentage.

### Clinical presentation

**Figure 1:**
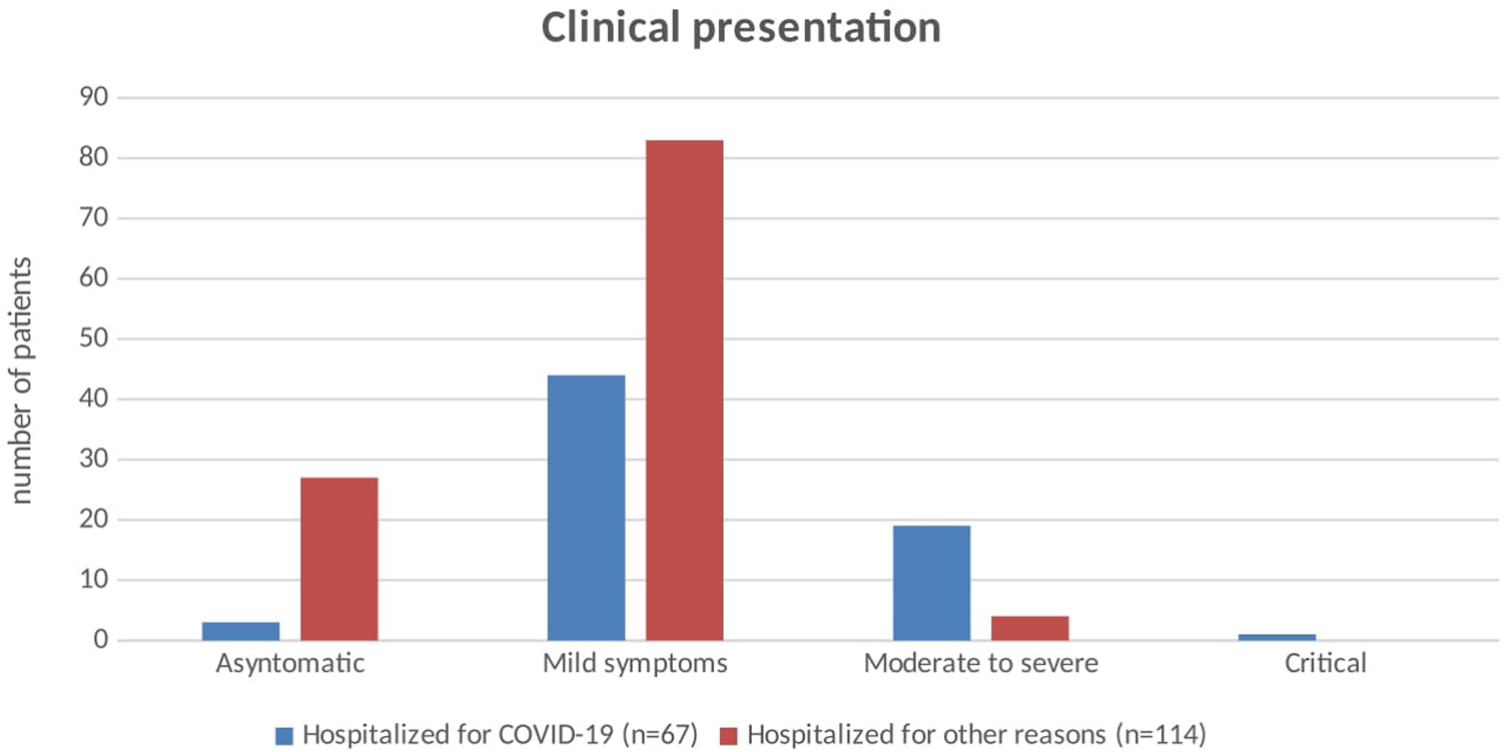
Clinical presentation of SARS-CoV-2 infection in children hospitalized for COVID-19 and in those hospitalized for other reasons. SARS-CoV-2, severe acute respiratory syndrome coronavirus 2; COVID-19, coronavirus disease 2019.

Clinical presentations of SARS-CoV-2 infection are reported in Table 2 and Figure 1. Children admitted for COVID-19 had mild to moderate and even critical symptoms while those hospitalized for other reasons were more often asymptomatic or presented mild symptoms. Hyperpyrexia was the most frequently reported symptom, especially at the onset of the disease.

**Table 2:** Clinical manifestations in children hospitalized for SARS-CoV-2 infection and in those admitted for other reasons and with an incidental diagnosis of SARS-CoV-2 infection.

Symptoms	Children hospitalized for COVID-19 (*N* = 67)	Children admitted for other reasons with incidental diagnosis of SARS-CoV-2 infection (*N* = 114)
	*n* (%)	*n* (%)
Fever	50 (74.6)	62 (54.3)
Cough	34 (50.7)	14 (12.3)
Coryza	6 (8.9)	11 (9.6)
Sore throat	5 (7.4)	13 (11.4)
Conjunctivitis	3 (4.5)	2 (1.8)
Dyspnea	14 (20.9)	5 (4.4)
Vomiting	11 (29.7)	26 (22.8)
Diarrhea	15 (22.3)	19 (16.6)
Abdominal pain	11 (16.4)	23 (20.1)
Smell and taste dysfunction	1 (1.5)	1 (0.8)
Skin rash	5 (7.5)	9 (7.9)
Seizures	0	17 (14.9)
Arthromyalgia	0	5 (4.4)
Chest pain	0	3 (2.6)

SARS-CoV-2, severe acute respiratory syndrome coronavirus 2; COVID-19, coronavirus disease 2019.

The first number is the absolute value, while the second, in brackets, is the associated percentage.

In children under 12 months of age, the second most represented symptom, after fever, was diarrhea. In the age group of 1–6 years, fever confirmed to be the main symptom, but the number of patients with respiratory symptoms increased. Finally, in the age group >11 years, cough was reconfirmed as the main symptom, but adolescents presented symptoms such as arthromyalgia, anosmia, ageusia and chest pain, which were absent or infrequent in the other age groups (Supplementary Figure S2).

### Coinfections

Concomitant infecting pathogens were searched in 135/181 (74.6%) patients: 75/114 among children were hospitalized for other reasons than SARS-CoV-2 infection and 60/67 among children were admitted for COVID-19; a coinfection was found in 40/75 (53.3%) in the first group and 32/60 (53.3%) in the second one. Viruses were the most frequently encountered pathogens (47/72 had almost one virus isolated on specimens); in particular, we reported 26 children with multiple infections, of which 19 with one or more viruses (PCR on nasopharyngeal aspirates, on feces or isolation on blood/antibodies). Rhinovirus was isolated in 31/47 nasopharyngeal aspirates performed. In 27 children, a bacterial infection was detected (stool culture, blood culture, urinary culture, or culture of specimens from the respiratory tract). Six children with a concomitant pathogen (on feces, blood, or respiratory sample) among those hospitalized for other reasons and eight among those admitted for COVID-19 developed complications; 7/14 had rhinovirus detected on nasopharyngeal aspirate.

### Biochemical blood analysis

**Figure 2:**
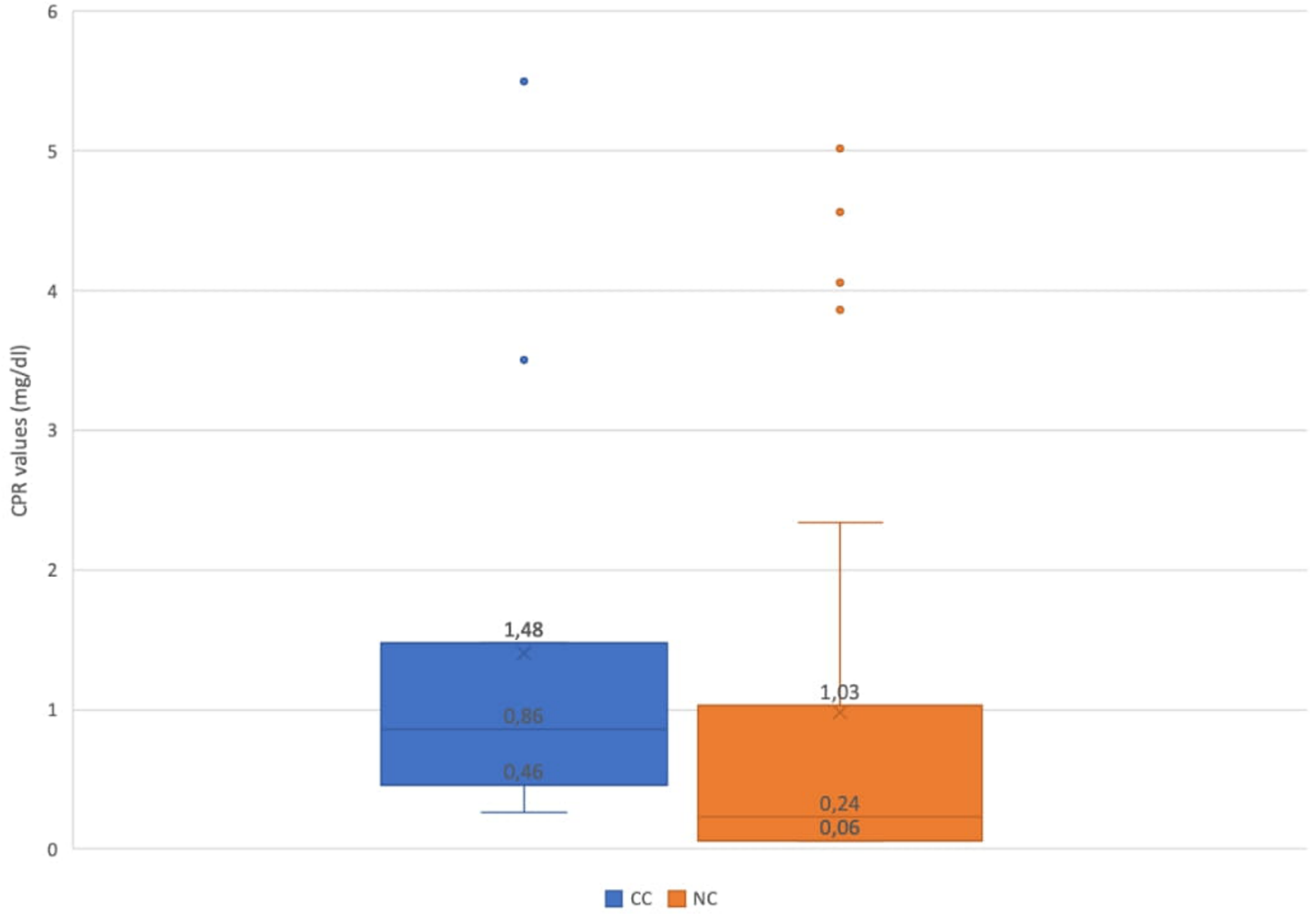
Comparison of C-reactive protein values of children with NC and CC. NC, noncomplicated COVID-19; CC, complicated COVID-19.

Children admitted for other reasons with an incidental diagnosis of SARS-CoV-2 infection were excluded from the biochemical analysis for the difficulty in discriminating the contribution in the results made by COVID-19 or by the true reason for admission. Among children hospitalized for SARS-CoV-2 infection, CRP value was increased (>0.05 mg/dL) in 41/67 patients (61.2%). After stratifying between children with NC and CC, a comparison of CRP values was performed; the mean value resulted higher in those with complications (*p-*value = 0.045). Specifically, in CC cases, the median value was 0.86 mg/dL (IQR 0.46–1.48), while in NC cases the median value was 0.24 mg/dL (IQR 0.06–1.03) (Figure 2).

Of 67 children, 14 had a low lymphocyte count; 7 of them belonged to the group of complicated COVID-19. An increased value of ferritin was observed in 14/67 children, while 13/67 patients had a high value of Lactate dehydrogenase (LDH) and 16/67 showed an elevation of D-dimer. No differences were detected among children with noncomplicated and complicated COVID-19.

### Outcomes and treatments

Overall, 27 patients developed respiratory complication related to SARS-CoV-2 infection; specifically, 8 out the 114 hospitalized for other reasons (7%) and 19 among the 67 children admitted for SARS-CoV-2 infection (28.3%) (Table 3). Only 2/8 patients of the first group (25%) required access to PICU for complications not related to COVID-19, one previously healthy and the other one with prematurity. All the patients of the second group who developed SARI (12/19–63.2%) and ARDS (1/19–5.3%) required oxygen supplementation and 10/19 (52.6%) needed respiratory support. One child, previously healthy, needed mechanical ventilation due to a rapid evolution to ARDS and required access to a PICU. Specific treatments (casirivimab + imdemivab; hydroxychloroquine and lopinavir/ritonavir; remdesivir) were considered only in three patients: one child previously healthy, another one with trisomy 21, and one with congenital deafness. One child affected by cystic fibrosis died due to COVID-19 complications; while two patients were discharged with transitory sequelae. The description of complications, comorbidities, and treatments for the two subgroups are shown in Supplementary Tables S3A,B.

**Table 3:** Characteristics of children who developed complications and hospitalized with COVID-19 or admitted for other reasons and with incidental diagnosis of SARS-CoV-2 infection.

	Children hospitalized for COVID-19 (*N* = 19)	Children admitted for other reasons with incidental diagnosis of SARS-CoV-2 infection (*N* = 8)
	*n* (%)	*n* (%)
Age category (years)		
<1 year	4 (21.1)	2 (25)
<1 and ≤6	7 (36.8)	4 (50)
<7 and ≤10	2 (10.5)	1 (12.5)
>11	6 (31.6)	1 (12.5)
Gender		
Males	9 (47.3)	6 (75)
Females	10 (52.7)	2 (25)
Ethnicity		
Caucasian	12 (63.2)	7 (87.5)
Not Caucasian	7 (36.8)	1 (12.5)
Prematurity, yes	3 (15.7)	2 (25)
Underlying chronic diseases, yes	9 (47.4)	3 (37.5)
Symptoms		
Fever	12 (63.2)	8 (100)
Respiratory symptoms	16 (84.2)	4 (50)
Gastroenteric symptoms	9 (47.4)	4 (50)
Coinfection, yes	12 (63.2)	6 (75)
Complications		
Viral pneumonia	6 (31.6)	4 (50)
SARI without O_2_	0	2 (25)
SARI with O_2_	12 (63.1)	2 (25)
ARDS	1 (5.3)	0
Admission to PICU, yes	1 (5.3)	2 (25)
Specific treatment, yes	2 (10.5)	2 (25)
Sequelae, yes	3 (15.7)	2 (25)
Death, yes	1 (5.3)	0
Duration of hospitalization (days), median	6 (IQR 4–10)	5.5 (IQR 4–8.5)

SARS-CoV-2, severe acute respiratory syndrome coronavirus 2; COVID-19, coronavirus disease 2019; SARI, severe acute respiratory illness; ARDS, acute respiratory distress syndrome; PICU, pediatric intensive care unit; IQR, interquartile range.

The first number is the absolute value, while the second, in brackets, is the associated percentage.

Among those who were hospitalized but did not develop complications, none received specific treatment, and all recovered without sequelae.

The median duration of hospital stay was 4.0 (3–6) days for children admitted for COVID-19 and 7.0 (4–12) days for those hospitalized for other reasons. The median duration of hospitalization among children with COVID-19 was 6 days for those who developed complications and 4 days for the others. Children with comorbidities have a recovery longer than those previously healthy [7 (4–13) vs. 5 (3–9) days].

### Risk factors

**Figure 3:**
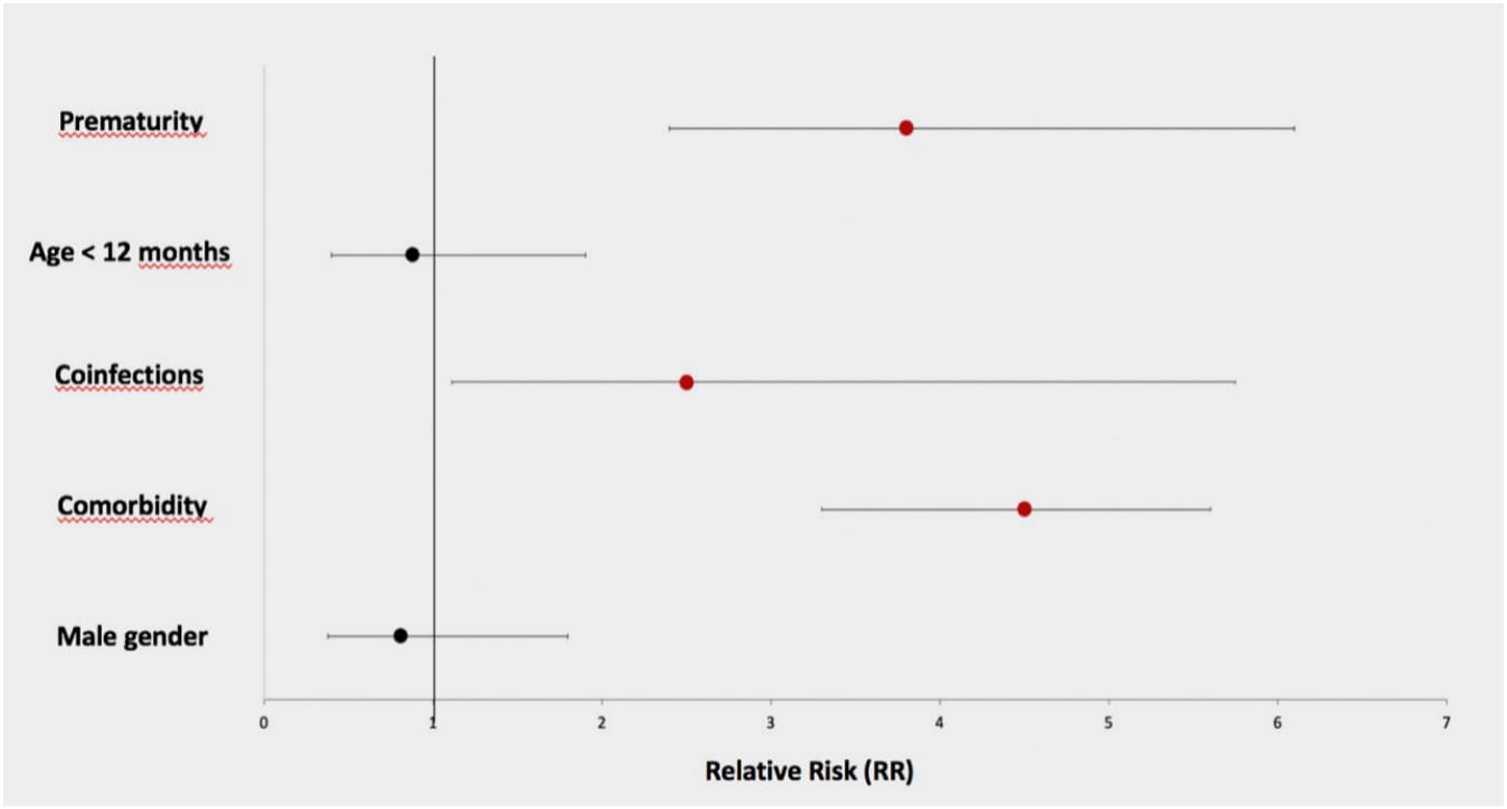
Relative risk for each risk factor.

Clinical risk factors associated with COVID-19 complications development were evaluated in the total children cohort (Figure 3). The main risk factors were prematurity (RR 3.8, 95% CI 2.4–6.1), comorbidities (RR 4.5, 95% CI 3.3–5.6), and coinfections (RR 2.5, 95% CI 1.11–5.75). On the contrary, male sex, an important risk factor in adults, was not confirmed in our population (RR 0.81, CI 0.38–1.8). In contrast to other studies, younger children (age <12 months) did not show an increased risk of evolution to complicated forms (RR 0.8, 95% CI 0.4–1.9).

### Vaccination status

From June 2021 to November 2021, when COVID-19 vaccines had only been approved for children >12 years old, 3 children of the 19 hospitalized were older than 12 years; only one had been vaccinated (with the first dose). From December 2021 to March 2022, the vaccination had been extended to children >6 years old. During this period, among the 24 children older than 6 years admitted to our department, only 14 had been vaccinated. Of the 15 vaccinated children, only one developed a COVID-related pneumonia and, specifically, in the week after the first dose of vaccine.

### Impact of genetic predisposition

The frequency distribution of the genetic variants analyzed is reported in Table 4A.

**Table 4A:** Frequency variant distribution of common genetic risk factors associated to COVID-19 susceptibility and severity.

	*OAS1/2/3* rs10735079 G > A		*DPP9* rs2109069 G > A		*IFNAR2* rs2229207 T > C		*LZTFL1* rs11385942 G > GA		*FUT2* rs601338 G > A		*ABO* rs657152 0 vs. non-0	
		%		%		%		%		%		%
Children cohort (*n* = 79)	GG	15.19	GG	53.16	TT	86.08	G	88.61	GG	36.71	0	41.77
	GA	40.51	GA	37.97	TC	13.92	GGA	10.31	GA	49.37	non-0	58.23
	AA	44.3	AA	8.86	CC	0	GAGA	1.27	AA	13.92		
Control cohort (*n* = 2,848)	GG	14.43	GG	54.11	TT	81.64	G	82.27	GG	29.42	0	39.68
	GA	48.35	GA	38.27	TC	16.92	GGA	16.92	GA	51.19	non-0	60.32
	AA	37.22	AA	7.62	CC	1.44	GAGA	0.81	AA	19.38		
COVID-19 adult cohort (*n* = 995)	GG	13.47	GG	47.34	TT	80	G	74.67	GG	30.45	0	35.18
	GA	47.14	GA	42.01	TC	18.49	GGA	23.92	GA	48.34	non-0	64.82
	AA	39.4	AA	10.65	CC	1.51	GAGA	1.41	AA	21.21		
*p*-value children vs. control		0.41		0.76		0.23		0.2		0.1		0.58
*p*-value children vs. adult		0.68		0.32		0.14		**0** **.** **01**		0.1		0.24
*p*-value adult vs. control		0.21		**0** **.** **0001**		0.28		**0** **.** **0001**		0.75		**0** **.** **01**

COVID-19, coronavirus disease 2019

Bold values are statistically significant p-value.

While *DPP9*, *LZTFL1*, and *ABO* risk variants were significantly more represented in adult patients with severe COVID-19 than in healthy controls, no significant differences were detected between frequency distribution of genetic variants between SARS-CoV-2-positive children and healthy controls. Interestingly, comparing frequency distribution between children and adult COVID-19 cohort, a significant difference in *LZTFL1* frequency was detected, being the high-risk allele less frequent in children COVID-19 cohort with respect to adult COVID-19 cohort, characterized by more severe respiratory involvement.

Subsequently, we stratified the children cohort according to the development of SARS-CoV-2 infection complications; a comparison between frequency variants distribution in children with NC vs. CC was performed. Noteworthy, all the children who developed a complication after SARS-CoV-2 infection carried the *OAS1/2/3* risk variant, both in heterozygous or homozygous manner (Table 4B).

**Table 4B:** Frequency variant distribution of common genetic risk factors associated to COVID-19 susceptibility and severity in children cohort stratified according to the development of SARS-CoV-2 infection complications.

	*OAS1/2/3* rs10735079 G > A		*DPP9* rs2109069 G > A		*IFNAR2* rs2229207 T > C		*LZTFL1* rs11385942 G > GA		*FUT2* rs601338 G > A		*ABO* rs657152 0 vs. non-0	
		%		%		%		%		%		%
Children with complicated COVID-19 (CC *n* = 15)	GG	0	GG	33.33	TT	86.67	G	86.67	GG	46.67	0	46.67
	GA	46.67	GA	53.33	TC	13.33	GGA	13.33	GA	40	non-0	53.33
	AA	53.33	AA	13.33	CC	0	GAGA	0	AA	13.33		
Children with noncomplicated COVID-19 (NC *n* = 64)	GG	18.75	GG	57.81	TT	85.93	G	89.06	GG	34.37	0	40.62
	GA	39.06	GA	34.37	TC	14.06	GGA	9.37	GA	51.56	non-0	59.37
	AA	42.180	AA	7.81	CC	0	GAGA	1.560	AA	14.06		
*p*-value CC vs. NC		0.15		0.11		0.94		0.93		0.5		0.66

SARS-CoV-2, severe acute respiratory syndrome coronavirus 2; COVID-19, coronavirus disease 2019.

We then evaluated the associations between genetic risk variants and the development of clinical adverse outcome of SARS-CoV-2 infection in the pediatric cohort. Specifically, through a univariate regression analysis, we evaluated the association between risk allele carrier status for each genetic variant (*OAS1/2/3*, *DPP9*, *IFNAR2*, *LZTFL1*, *ABO*, and *FUT2*) and the development of clinical complications and of pneumonia. No significant associations were detected between risk alleles carrier status and the development of clinical complications; however, the *OAS1/2/3* variant tended to be associated with a higher risk of pneumonia (OR 2.9, 95% CI 0.95–8.88; *p-*value 0.059) (Table 4C). We then performed a multivariate regression analysis to dissect the role of different genetic and clinical risk factors on the main clinical outcomes and to evaluate their relative role. The association between *OAS1/2/3* variant and pneumonia development was statistically significant after adjusting for the main genetic factors known to predispose to severe COVID-19: *LZTFL1* and *DPP9* variants, and remained significant also after adjusting for the main clinical risk factors identified in children cohort (presence of chronic diseases and prematurity) (OR 3.28, 95% CI 1–10.7; *p-*value 0.049). Therefore, *OAS1/2/3* variation, tagged by rs10735079, was associated with increased risk for pneumonia, independent of other genetic or clinical risk factors (Table 4D). The association was also confirmed comparing *OAS1/2/3* variant frequency in the children cohort with pneumonia to that of the control cohort (OR 3.2; 95% CI 1.08–9.4; *p-*value 0.035).

**Table 4C:** Univariate logistic model of genetic factors associated with clinical complication or pneumonia development in children cohort.

Genetic variable	Clinical complications			Pneumonia		
	OR	95% CI	*p*	OR	95% CI	*p*
*OAS1/2/3* rs10735079. allele A	1.91	0.78–4.66	0.15	2.9	0.95–8.88	0.059
*DPP9* rs2109069. allele A	1.94	0.84–4.46	0.11	1.65	0.67–4.05	0.27
*IFNAR2* rs2229207. allele C	0.94	0.18–4.88	0.94	1.28	0.24–6.86	0.76
*LZTFL1* rs11385942. allele GA	1.06	0.23–4.73	0.93	1.36	0.3–6.1	0.68
*ABO* rs657152. allele C	0.91	0.39–2.16	0.84	0.92	0.36–2.35	0.86
*FUT2* rs601338. allele A	0.74	0.31–1.75	0.5	0.94	0.37–2.35	0.9

**Table 4D:** Multivariate logistic model of genetic and clinical factors associated with pneumonia development in children cohort.

Variable	Adjusted model		
	OR	95% CI	*p*
*OAS1/2/3* rs10735079. allele A	3.28	1–10.8	**0** **.** **049**
*DPP9* rs2109069. allele A	1.69	0.6–4.8	0.31
*LZTFL1* rs11385942. allele GA	1.46	0.2–8.5	0.67
Chronic diseases. yes	3.5	0.9–14.6	0.07
Prematurity. yes	2.27	0.2–21.3	0.47

## Discussion

Our study shows that COVID-19 is less severe in the pediatric population, even if complications can develop, especially in those children with comorbidities (chronic disease or prematurity) and with coinfections. In our case series, those hospitalized for other reasons were 114/181 (62%), a rate slightly higher than the results registered by Webb and Osburn. and Kushner et al. in their case series (40%–45% of accidental positivity to SARS-CoV-2) (24, 25).

Admission for reasons other than COVID-19 was higher in all the three waves, with a progressively increasing trend for COVID-19-related ones: the number of hospitalizations related to SARS-CoV-2 was probably underestimated during the first months of the pandemic due to the lowered knowledge of the possible clinical manifestations in the pediatric age. Children had frequently a contact history with infected households: in our cohort, 40% of children had one parent positive for SARS-CoV-2 (11). The mean age of children hospitalized with and/or due to SARS-CoV-2 infection was 5.7 years. The male gender resulted in the most hospitalized, but male sex was not associated with more severe illness. The group age most hospitalized was infants <12 months in line with what has been reported in the literature but with a percentage lower than that described in other studies, probably because newborns are not admitted to our ward. Among infants <12 months of age, less than 10% developed complications (of which most had comorbidities): such a low complication rate suggests a prudent attitude resulting from the few months of life and not related to the severity of illness, as confirmed by the result of the RR.

In the present case series, comorbidities were reported in the 30% of children admitted with/for SARS-CoV-2 infection, a rate higher than others reported in the literature (11). Prudent behavior may have influenced the decision to admit a SARS-CoV-2-positive patient only because of the chronicity, even if the comorbidities such as chronic diseases or prematurity were found to be factors that increased the risk of developing complications. The length of stay was higher in children admitted for reasons other than COVID-19, because they were identified in the emergency department as requiring hospitalization (for severe infections such as pyelonephritis, osteomyelitis, or for relapses of chronic diseases) and SARS-CoV-2 was detected incidentally on admission. Other factors contributing to the different length of stay are the heterogeneity of the sample, which was numerically larger and with a higher proportion of children affected by chronic diseases.

Regarding clinical features, in all age groups, the most frequent symptom was fever followed by cough (Supplementary Figure S2). However, a high incidence of gastrointestinal symptoms such as vomiting, diarrhea, and abdominal pain is observed in the pediatric age group with a higher incidence than in the adult–elderly age group (26). The involvement of gastrointestinal tract seems to be more frequent in children, but the reason is unclear. One possibility is the different expression on enterocytes of angiotensin-converting enzyme 2 and transmembrane protease serine 2, two receptors essential for the cellular entry process of the virus. The virions released in the gastrointestinal tract disrupt enterocytes with the appearance of symptoms and viral shedding in the stools, suggesting the reason of the detections of SARS-CoV-2 in the feces of the affected. There is also an alteration of the intestinal microbiome contributing in digestive symptoms (26, 27). In contrast, symptoms such as dyspnea, anosmia, ageusia, and arthromyalgia are occasional, especially in younger children (28). Data reported in our study confirmed that asymptomatic children and children with mild disease (87.3%) are the most represented.

Within our cohort, the complication rate was 14.9%, comparable to findings described in the multicenter study by Garazzino et al. where a rate of 16% was reported; complications were higher in those admitted for SARS-CoV2 infection than those who incidentally tested positive for SARS-CoV-2 (11). Among chronic patients hospitalized for SARS-CoV-2 infection, 47.3% developed complications, while among previously healthy patients, only 20.8% experienced complications. Among those who developed complications, the number of subjects with coinfections was also higher. The role of both comorbidities (chronic disease and prematurity) and coinfections as risk factors for the development of complications is reconfirmed (29). As in the adult age, respiratory complications were the most frequent; in the other cases neurological, gastrointestinal or infectious complications occurred. Among patients with pulmonary complications, 16/27 developed SARI, while only one evolved to ARDS. Once again, we thus confirm a low incidence and severity of complications in pediatric age when compared with an adult hospital setting. Moreover, in our cohort, vaccinated children were found to be at lower risk of developing complications, although our sample size is too small to provide statistically significant results.

Regarding the role of genetic risk factors for severe COVID-19, we did not observe significant differences in frequency distribution in affected children vs. the general population. Interestingly, the frequency of chromosome 3 risk allele was significantly lower in SARS-CoV-2-positive children reported in the present cohort compared to adults with severe respiratory disease admitted at the same hospital. In contrast, previous studies suggested that genetic risk variants for severe COVID-19, in particular, those at the chromosome 3 gene cluster had a larger impact in younger vs. older individuals (16). These data suggest that the described genetic risk variants at *LZTFL1* and *DPP9* loci are mainly risk factors for respiratory failure and not for the risk of severe infection (30).

The *OAS1/2/3* risk allele frequency in the pediatric cohort was comparable to that of the adult cohort and the general population; however, after stratification of the pediatric cohort according to the development of infection complications, the *OAS1/2/3* risk allele was more represented in children with complications vs. children without complications and local healthy controls. Furthermore, *OAS1/2/3* variation was associated with the risk of developing pneumonia, after adjusting for the other genetic risk factors associated with severe COVID-19 and the main clinical factors associated to complications development in children. These data suggest that in SARS-CoV-2-positive children, *OAS1/2/3* genes cluster is the main genetic risk factor predisposing to pneumonia, independent of other genetic and clinical factors. The causal variant underlying the association of the *OAS1/2/3* locus with the susceptibility to severe COVID-19 is likely represented by rs10774671, encoding for a splice-acceptor variant of OAS1, where the protective allele determines the synthesis of a more active prenylated form of OAS1, involved in the intracellular innate immune responses against RNA-viruses, with a capacity for membrane localization (19).

This is the first single-center study conducted for 24 consecutive months on SARS-CoV-2 infection in hospitalized children. Another strength of our study is the differentiation between those who were admitted for or with SARS-CoV-2 positivity and the focus we placed on clinical and genetic risk factors that increase the likelihood of developing complications.

A major limitation of our study is that our hospital is a referral center for chronic diseases (kidney diseases, cystic fibrosis, metabolic disorders), so the number of patients admitted with comorbidities may be overestimated. On the other hand, since our hospital is not a referral for oncological diseases or endocrinological diseases (obesity or diabetes), the type of comorbidities reported are influenced and differ from what other studies have reported in literature (13). Another limitation is the lack of data on newborns with SARS-CoV-2 infection who are usually admitted to the neonatology unit and not to our pediatric department.

In conclusion, our study confirms that most children are hospitalized for reasons unrelated to SARS-CoV-2 and experienced an asymptomatic or clinically mild infection with no need for specific therapies and with complete resolution without long-term sequelae. Gastrointestinal symptoms occur more frequently than in adults. In those who were hospitalized due to the infection, the risk of developing complications was higher, especially in those with comorbidities (prematurity and chronic disease) or in whom coinfection was identified. The most frequent complication was pneumonia with SARI, whereas evolution to ARDS was rare. The *OAS* gene cluster, involved in innate antiviral defenses and coding for enzymes degrading viral dsRNA, seem to be a major genetic risk factor in children. Polymorphisms in *OAS* gene cluster have been previously associated with predisposition to different viral infection and variations in clinical outcomes (31, 32). It could be speculated that children carrier of *OAS* risk allele could be more prone to develop viral infections, also different from SARS-CoV-2, so that the more adverse outcomes of SARS-CoV-2 infection observed in these children could be related to a more susceptibility to develop coinfections. All in all, despite studies in larger and independent cohorts are needed to dissect the role of clinical and genetic risk factors, results highlight possible differences in the genetic architecture in the susceptibility to develop severe COVID-19 between children and even young adults.

## Data Availability

The data presented in the study are deposited in the European Variation Archive repository. Accession number project: PRJEB58778, Analyses: ERZ15578599. Link: https://www.ebi.ac.uk/ena/browser/view/PRJEB58778.
