# Supplementary material for: SARS-CoV-2 infection in children: A 24 months experience with focus on risk factors in a pediatric tertiary care hospital in Milan, Italy
Source: Front Pediatr. 2023 Feb 17;11:1082083. doi: 10.3389/fped.2023.1082083 (PMC9981971; doi:10.3389/fped.2023.1082083)
Supplement: Supplementary file 1 [file Table1.pdf]

Supplementary Material

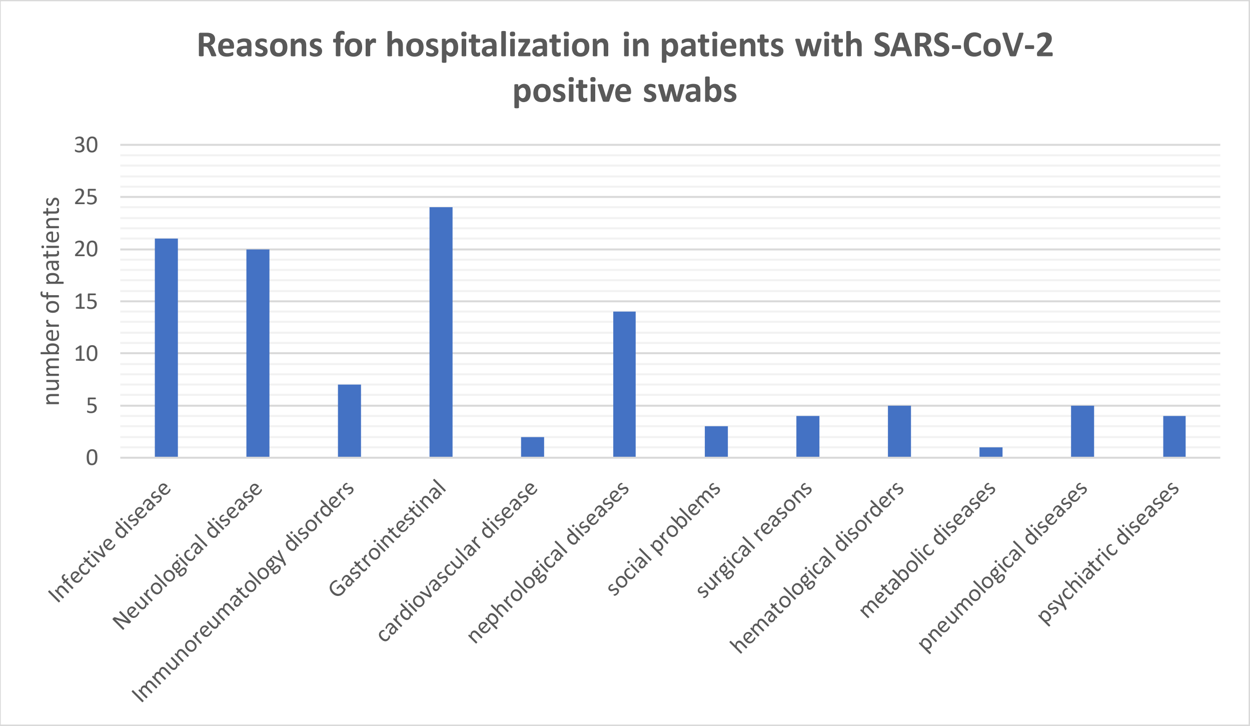

Supplementary Figure 1. Reasons for hospitalization in children with an incidental diagnosis of SARS-CoV-2 infection.

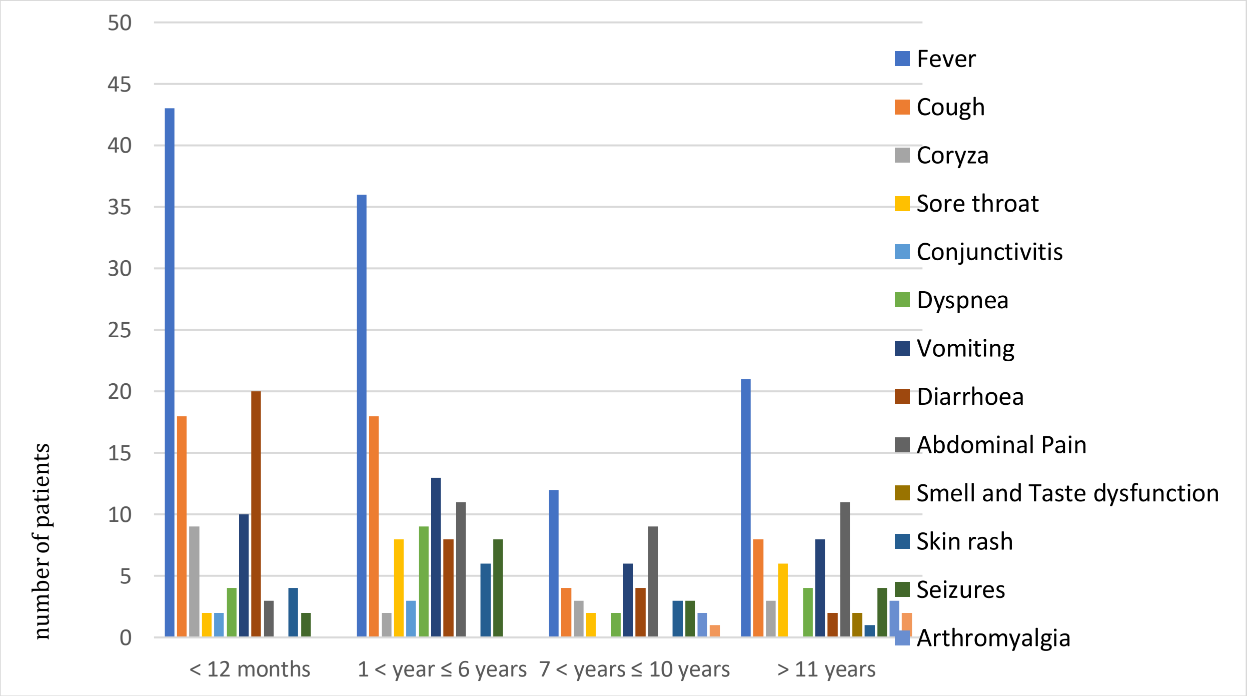

Supplementary Figure 2. COVID-19 related clinical manifestations according to age.

	Number of patients (Total=19/67)		Comorbidities (9/19)		PICU (1/19)	Specific treatment (2/19)		Death (1/19)	Sequelae (2/19)
**Viral pneumonia without respiratory failure**	6	(2/9 with O_2 supplementation)	2/6	(Pyloric stenosis, obesity)	0	0		0	0
**SARI without 0_2**	0		0		0	0		0	0
**SARI with 0_2**	12	9/12 needed CPAP or High Flow nasal cannula	7/12	(Pulmonary broncho dysplasia (3), congenital deafness, cystic fibrosis, Down syndrome, metabolic disorded)	0	2	Remdesivir; Casirivimab + Imdevimab	1 (affected by cystic fibrosis)	2 (Transitory increase of FiO2 at home high-flow nasal cannula; signs of pneumonia at the control X-ray two month later)
**ARDS**	1	(1/1 O_2 supplementation)	0		1	0		0	0

Supplementary Table 3A. Characteristics of children hospitalized for COVID-19 who developed complications (viral pneumonia, SARI with 0_2, SARI without 0_2 and ARDS). For each complication, patient number, co-morbidities, need for specific treatments, and possible death or long-term sequelae are listed.

	Number of patients (Total=8/114)		Comorbidities (3/8)		PICU (2/8)		Specific treatment (1/8)		Death (0/8)	Sequelae (0/8)
**Viral pneumonia without respiratory failure**	4	(0/4 with O_2 supplementation)	2/4	(Heart disease, deafness)	1	For reason different than COVID-19	1	(lopinavir/ ritonavir + Hydroxiclorochine)	0	0
**SARI with 0_2**	3		2/3	One with prematurity but healthy and another one with problems related to prematurity	1	For reason different than COVID-19	0		0	0
**SARI without 0_2**	1		0/1	With prematurity but healthy	0		0		0	0
**ARDS**	0		0		0		0		0	0

Supplementary Table 3B. Characteristics of children hospitalized for other reasons than COVID-19 and who developed complications (viral pneumonia, SARI with 0_2, SARI without 0_2 and ARDS). For each complication, patient number, co-morbidities, need for specific treatments, and possible death or long-term sequelae are listed.
